# Full thickness macular hole case after intravitreal aflibercept treatment

**DOI:** 10.1186/s12886-015-0021-3

**Published:** 2015-03-29

**Authors:** Yuji Oshima, Rajendra S Apte, Shintaro Nakao, Shigeo Yoshida, Tatsuro Ishibashi

**Affiliations:** Department of Ophthalmology, Graduate School of Medical Sciences, Kyushu University, 3-1-1 Maidashi, Higashi-ku, Fukuoka, 812-8582 Japan; Department of Ophthalmology and Visual Sciences, Washington University School of Medicine, St. Louis, MO USA

**Keywords:** Age-related macular degeneration, Tear of retinal pigment epithelium, Macular hole, Aflibercept

## Abstract

**Background:**

The pathogenesis of macular hole formation is widely accepted as a tractional force at the vitreo-retinal interface in fovea. We report a case of macular hole after intravitreous aflibercept injection for age-related macular degeneration (AMD) associated with contraction of the retinal pigment epithelium (RPE) at the edge of a fibrovascular pigment epithelial detachment (PED).

**Case presentation:**

A 94-year old man with neovascular AMD affecting his left eye accompanied by a fibrovascular PED was examined for severe vision loss. Although RPE tear in his left eye was identified before the first aflibercept intravitreous injection performed in order to treat neovascular AMD, he received three aflibercept injections as induction treatment. After induction treatment, a full thickness macular hole was identified associated with the contracted rolled RPE edge beneath the retina.

**Conclusion:**

Macular hole is commonly formed associated with tangential vitreous traction. Current report suggests that rapid contraction of the RPE underneath the retina can be one of the causes of a macular hole, and one of the side effects of anti-VEGF therapy for neovascular AMD.

## Background

Neovascular Age-Related Macular Degeneration (wet AMD) is the major cause of legal blindness among the elderly people in many countries. Anti-vascular endothelial growth factor (VEGF) therapy is now the major treatment in order to prevent severe vision loss and possibly improve vision. Tears of the retinal pigment epithelium (RPE) are recognized as complication of wet AMD in patients with pigment epithelial detachments (PED) [1]. Although several recent reports indicated that anti-VEGF antagonists, such as pegaptanib, bevacizumab, and ranibizumab might increase the risk of tears of RPE compared to natural course [2-5]. Cunningham et al. reported that there aren’t significantly differences of the incident of RPE tear after ranibizumab treatment for wet AMD patients in ANCHOR, MARINA, and PIER study compared to natural course [6]. Idiopathic macular holes are full-thickness retinal defects in the foveal neurosensory retina. The pathogenesis of macular hole formation is widely accepted as an abnormal anteroposterior and tangential vitreous traction performed in foveal retinal surface [7]. We present a recent case of macular hole after intravitreous aflibercept injection associated with contraction of the RPE at the edge of a PED.

## Case presentation

**Figure 1:**
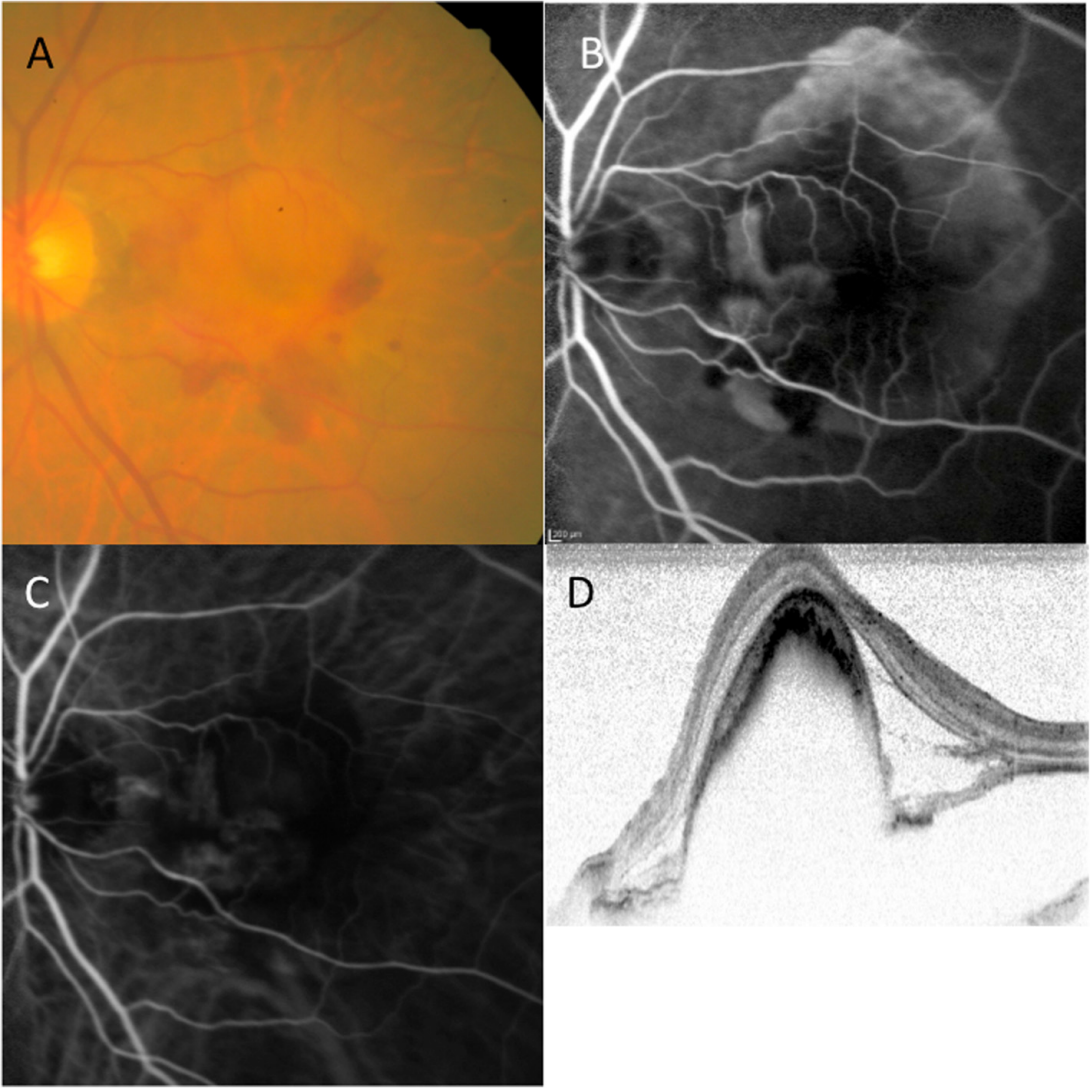
**Macular findings before treatment.** A 94-year-old man was treated with intravitreal aflibercept injection. At baseline, the best-corrected visual acuity was 1.7 log MAR. **(A)** Color fundus photograph shows subretinal hemorrhage, serous retinal detachment (SRD), and pigment epithelial detachment (PED). **(B)** Fluorescein angiography image showing leakage due to occult CNV and staining due to vascularized PED, and hyperfluorescence due to RPE tear. **(C)** Indocyanine green angiography image shows no underlying polypoidal lesion. **(D)** Optical coherence tomography (OCT) image showing PED and SRD.

**Figure 2:**
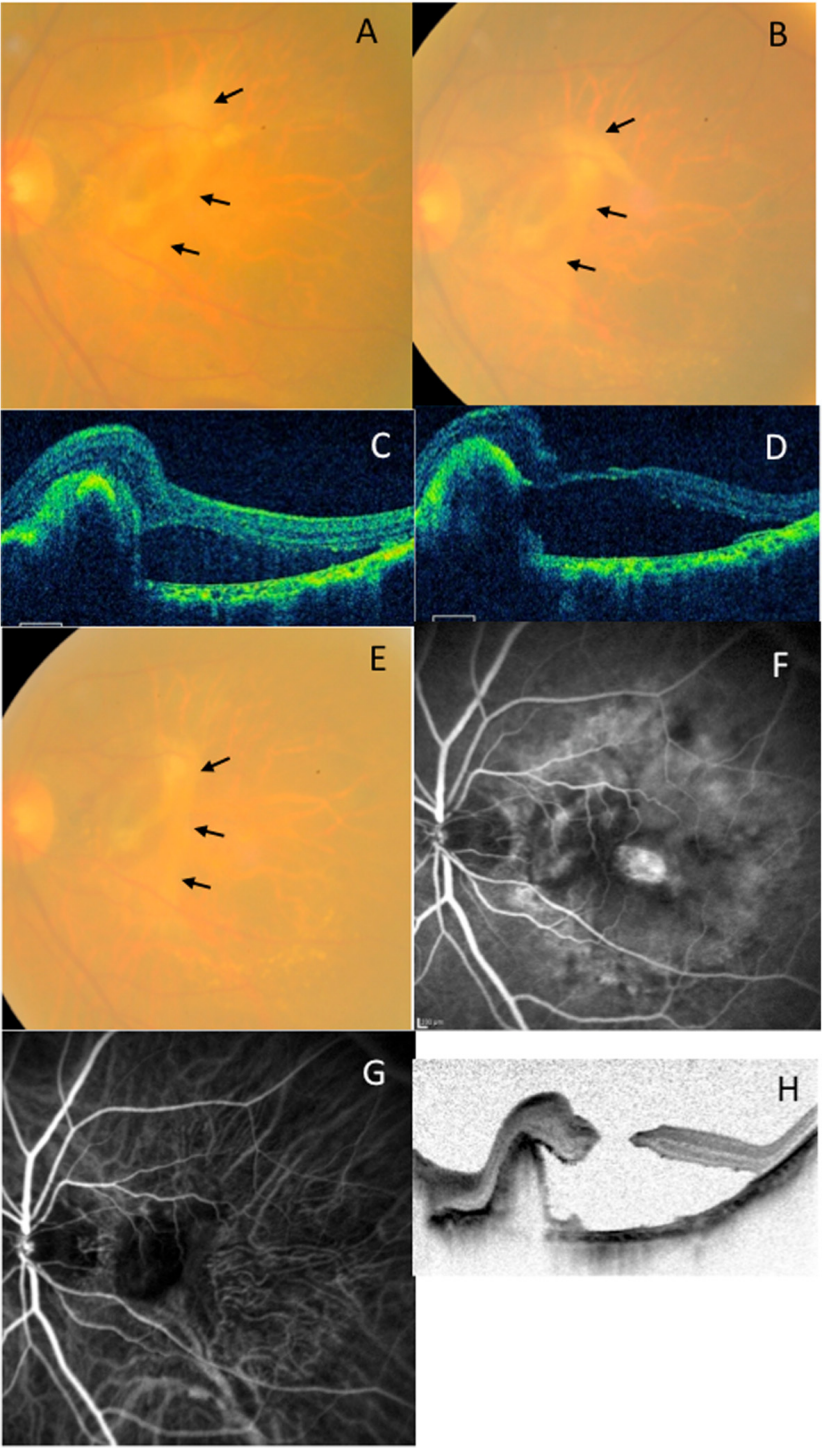
**The progression findings for macular hole formation. (A,C)** One month after first aflibercept injection. The color fundus photograph shows a tear of the RPE, rolled RPE flap beneath the retina, and subretinal fibrosis. OCT shows rolled RPE flap and SRD. **(B,D)** One month after second aflibercept injection. The color fundus photograph shows contracted subretinal fibrosis. OCT shows thin foveal retina secondary to traction associated with the rolled RPE flap. **(E, F,G, H)** One month after third aflibercept injection. The color fundus photograph shows contracted subretinal fibrosis and RPE tear **(E)**. Fluorescein angiography shows choroidal flush due to a macular hole **(F)**. Indocyanine green angiography shows blocked fluorescence due to subretinal fibrosis **(G)**. OCT shows full thickness macular hole with shrunken, rolled RPE flap **(H)**. Arrows show the edge of rolled RPE flap **(A, B, E)**.

A 94-year-old man presented with sudden visual disturbance of his left eye. At the first visit, the best corrected visual acuity (BCVA) in the right eye was 0.046 log MAR and in the left eye was 1.7 log MAR. The axial length of the right eye was 23.46 mm, and that of left eye was 23.47 mm. Fundus examination showed multiple drusen in the right eye, and subretinal bleeding and vascularized PED associated with wet AMD in the left eye. Fluorescein (FA) and Indocyanine (IA) angiography showed occult with no classic choroidal neovascularization (CNV) in the fovea and an RPE tear at the edge of PED. OCT showed a large PED and serous neurosensory retinal detachment. (Figure 1) After the first aflibercept intravitreous treatment, tear of the RPE and rolled RPE were clearly noted beneath the retina. OCT showed thinning of the central retina in the center of fovea stretched by the rolled and contracted RPE that was exacerbated after the second aflibercept injection. After the third injection performed as part of the induction treatment, the BCVA in the left eye was 1.7 log MAR, and full thickness macular hole formed due to stretched by rolled RPE contraction. (Figure 2) Observation was recommended because the patient did not desire any additional intervention. One year after first aflibercept treatment, fundus examination showed subretinal fibrosis, and full thickness macular hole remained open. The BCVA in the left eye was 1.7 log MAR, same as that seen at baseline.

## Discussion

RPE tear is well known as the complication of wet AMD, especially common in the patients with PED [8]. Although some reports suggested that anti-VEGF therapy might increase the risk of RPE tear as a complication of wet AMD treatment, subanalysis report of large scale prospective study revealed that the incidence of RPE tear after ranibizumab treatment is not significantly higher compared to natural course [6]. Tear of RPE as a complication has been reported with each different anti-VEGF agent, including bevacizumab, ranibizumab, pegaptanib, and aflibercept [2-4,9]. A recent retrospective study showed that the risk of RPE tear is significantly higher in patients with vascularized PED (vPED) than without PED, and shorter duration of PED and higher PED are significant risk factors [5]. The exact pathogenesis of RPE tear is unknown. But a recent report speculated that rapid involution and contraction of neovascular tissue adherent to the undersurface of the RPE may impart a substantial contractile force to tears [10]. The pathogenesis of macular hole formation is widely associated with anteroposterior vitreo-macular traction [11]. Although macular hole formation associated with AMD is rare, there have been reports AMD-associated macular hole development [12-15]. Okamoto et al. reported that traction of epiretinal membrane caused by exudative changes derived from CNV is supposed to be one of the reason for macular hole formation accompanied by AMD [14]. Raiji et al. reported full thickness macular hole formation overlying PED after intravitreous ranibizumab injection. They speculated the etiology as the tractional forces of vitreomacular adhesion and pushing or stretching forces of the choroidal neovascular complex may contribute to macular hole formation [15].

In this case, the patient received aflibercept injections for his wet AMD with vascularized PED. Tear of RPE at the edge of the PED occurred before the first injection and the rolled RPE flap involuted underneath the retina. Rolled RPE flap associated with subretinal fibrosis resulted in traction on the fovea from underneath the retina during aflibercept treatment. The contracted fovea above and beneath retina was split and there was progression to a full thickness macular hole. He didn’t receive surgical procedure for closing macular hole, because the macular hole was over an area devoid of RPE due to the RPE tear and that surgical closure of the hole would not likely lead to improvement in vision. His BCVA remained stable during further observation.

To our knowledge, this is the first case report of macular hole formation secondary to an RPE tear and contraction of the RPE edge and subretinal fibrosis following multiple intravitreal aflibercept injections. Although the incidence of full thickness macular hole formation, as a complication after anti-VEGF therapy is low, one should be mindful of such complications associated with anti-VEGF injections.

## Conclusion

Macular hole is commonly formed associated with tangential vitreous traction in the fovea. Current report suggests that rapid contraction of the RPE underneath the retina can be one of the causes of a macular hole, and one of the side effects of anti-VEGF therapy for neovascular AMD.

## Patient consent

Written informed consent was obtained from the patient for publication of this case report and any accompanying images. A copy of the written consent is available for review by the editor of this journal.

## Abbreviations

AMD: Age-related macular degeneration
CNV: Choroidal neovascularization
FA: Fluorescein angiogram
IA: Indocyanine green angiogram
RPE: Retinal pigment epithelium
PED: Pigment epithelial detachments
